# SSRE: Cell Type Detection Based on Sparse Subspace Representation and Similarity Enhancement

**DOI:** 10.1016/j.gpb.2020.09.004

**Published:** 2021-02-27

**Authors:** Zhenlan Liang, Min Li, Ruiqing Zheng, Yu Tian, Xuhua Yan, Jin Chen, Fang-Xiang Wu, Jianxin Wang

**Affiliations:** 1 School of Computer Science and Engineering, Central South University, Changsha 410083, China; 2 College of Medicine, University of Kentucky, Lexington, KY 40536, USA; 3 Division of Biomedical Engineering, University of Saskatchewan, Saskatoon, SK S7N 5A9, Canada

**Keywords:** Single-cell RNA sequencing, Clustering, Cell type, Similarity learning, Enhancement

## Abstract

Accurate identification of **cell types** from **single-cell RNA sequencing** (scRNA-seq) data plays a critical role in a variety of scRNA-seq analysis studies. This task corresponds to solving an unsupervised **clustering** problem, in which the similarity measurement between cells affects the result significantly. Although many approaches for cell type identification have been proposed, the accuracy still needs to be improved. In this study, we proposed a novel single-cell clustering framework based on **similarity learning**, called SSRE. SSRE models the relationships between cells based on subspace assumption, and generates a sparse representation of the cell-to-cell similarity. The sparse representation retains the most similar neighbors for each cell. Besides, three classical pairwise similarities are incorporated with a gene selection and **enhancement** strategy to further improve the effectiveness of SSRE. Tested on ten real scRNA-seq datasets and five simulated datasets, SSRE achieved the superior performance in most cases compared to several state-of-the-art single-cell clustering methods. In addition, SSRE can be extended to visualization of scRNA-seq data and identification of differentially expressed genes. The matlab and python implementations of SSRE are available at https://github.com/CSUBioGroup/SSRE.

## Introduction

With the recent emergence of **single-cell RNA sequencing** (scRNA-seq) technology, numerous scRNA-seq datasets have been generated, which brings unique challenges for advanced omics data analysis [1], [2]. Unlike bulk sequencing averaging the expression of mass cells, scRNA-seq technique quantifies gene expression at the single-cell resolution. Single-cell techniques promote a wide variety of biological topics such as cell heterogeneity, cell fate decision, and disease pathogenesis [3], [4], [5]. Among all the applications, cell type identification plays a fundamental role and its performance has a substantial impact on downstream studies [6]. However, identifying cell types from scRNA-seq data is still a challenging problem. The traditional clustering methods cannot work well on scRNA-seq data because of the high noise rate and high dropouts [7]. Therefore, new efficient and reliable clustering methods for cell type identification are urgent and meaningful.

In recent studies, several novel clustering approaches for detecting cell types from scRNA-seq data have been proposed. Among these methods, cell types are mainly decided on the basis of learned cell-to-cell similarity. For example, single-cell interpretation via multikernel learning (SIMLR) [8] visualizes and clusters cells using multi-kernel similarity learning [9], which performs well on grouping cells. Shared nearest neighbor (SNN)-Cliq [10] firstly constructs a distance matrix based on the Euclidean distance, and then introduces the shared k-nearest neighbors (KNN) model to redefine the similarity. SNN-Cliq provides both the estimation of cluster number and the clustering results by searching for quasi-cliques. Moreover, Corr [11] defines the cell-pair differentiability correlation instead of computing primary (dis)similarity like Pearson correlation and Euclidean distance. RAFSIL [12] divides genes into multiple clusters, and makes dimension reduction on each gene cluster. Then, RAFSIL concatenates the informative features obtained from each gene cluster. Finally, RAFSIL applies the random forest to calculate the similarities for each cell recursively. Besides, nonnegative matrix factorization (NMF) determines the cell types in the latent space [13], while SinNLRR [14] and AdaptiveSSC [15] learn the similarity matrix with nonnegative low rank and sparse constraints. Instead of learning a specific similarity, some researchers have turned to use ensemble learning that focuses on the consensus of multiple clustering methods [16], [17].

Even though many approaches have been applied to cell type identification, most of them are sensitive to noise, especially for the high-dimensional data. They generally compute the similarity between two cells merely considering the gene expression of these two cells [18]. In this study, we developed SSRE, a novel method for cell type identification. It focuses on similarity learning, in which the cell-to-cell similarity is measured by considering more similar neighbors. SSRE computes the linear representation between cells based on sparse subspace theory, and thus generates a sparse representation of cell-to-cell similarity [19]. Moreover, motivated by the observations that each similarity measurement can represent data from a different aspect [16], [20], SSRE incorporates three classical pairwise similarities into similarity learning. In order to reduce the effect of irrelevant features and improve the overall accuracy, SSRE designs a two-step procedure, *i.e.*, 1) adaptive gene selection and 2) similarity enhancement. The experimental results show that when combined with spectral clustering, the learned similarities by SSRE can reveal the block structure of scRNA-seq data reliably. Also, the experimental results on ten real scRNA-seq datasets and five simulated scRNA-seq datasets show that SSRE achieves higher accuracy of cell type detection in most cases than the compared popular approaches. Moreover, SSRE can be easily extended to other scRNA-seq tasks such as differential expression analysis and data visualization.

## Method

### Framework of SSRE

**Figure 1:**
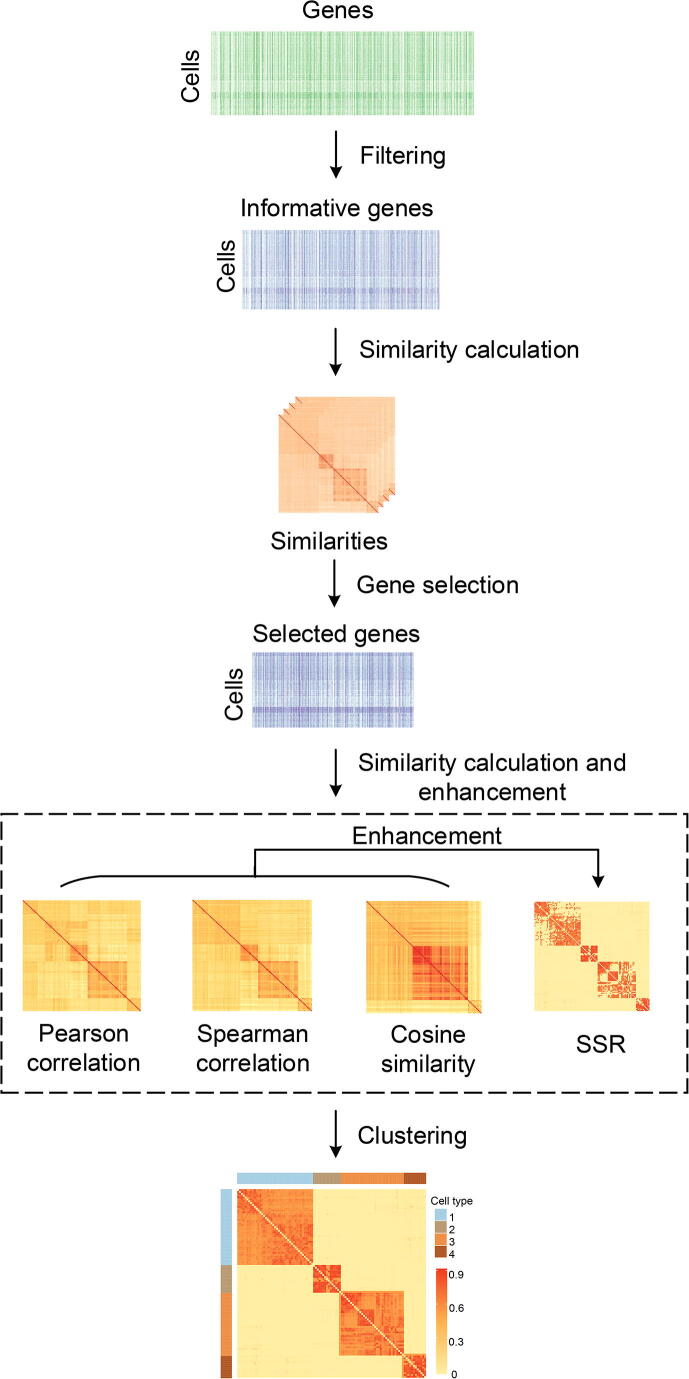
**The schematic diagram of SSRE** SSRE consists of five main parts, including gene filtering, similarity calculation, gene selection, similarity enhancement, and clustering. The original input is a gene expression matrix. After filtering, four similarity measurements (Pearson correlation, Spearman correlation, cosine similarity, and SSR) are applied to select informative genes. The selected gene expression matrix is then used as input to the subsequent process for single-cell clustering. SSRE, single-cell clustering framework based on similarity learning; SSR, sparse subspace representation.

We introduce the overview of SSRE briefly. A schematic diagram of SSRE is shown in Figure 1, and detailed steps of SSRE are introduced later in this section. Given a scRNA-seq expression matrix, SSRE first removes genes whose expression levels are zero in all the cells. Then, the informative genes are selected based on the sparse subspace representation (SSR), Pearson correlation, Spearman correlation, and cosine similarity. With the preprocessed gene expression matrix, SSRE learns SSR for each cell simultaneously. Then, SSRE derives an enhanced similarity matrix from the learned SSR similarity and the other three pairwise similarities. Finally, SSRE uses the enhanced similarity to identify cell types and visualize data.

### Sparse subspace representation

The estimation of the similarity (or distance) matrix is a crucial step in clustering [8]. If the similarity matrix is well generated, it could be relatively easier to distinguish the cluster. In this study, we adopted sparse subspace theory [19] to compute the linear representation between cells and generate a sparse representation of the cell-to-cell similarity. Some subspace-based clustering methods have been successfully applied to computer vision field, and have been proved to be highly robust in corrupted data [21], [22]. For scRNA-seq data, the sparse representation of cell-to-cell similarity is measured by considering the linear combination of similar neighbors. This tends to catch global structure information and generate more reliable similarity than traditional similarity measurement. The specific calculation processes are described as follows.

Mathematically, a scRNA-seq dataset with p genes and n cells can be denoted as $X=\left[x_{1},x_{2},\cdots ,x_{n}\right]\in R^{p\times n}$, where $x_{i}={\left[x_{i1},x_{i2},\cdots ,x_{\mathrm{ip}}\right]}^{T}$ indicates the expression profiles of the p genes in cell i. Its linear representation coefficient matrix $C=\left[c_{1},c_{2},\cdots ,c_{n}\right]\in R^{n\times n}$ satisfies the equation X=XC. According to the assumption that the expression of a cell can be represented by other cells in the same type, only the similarity of cells in the same cluster is non-zero. It also means that the coefficient matrix C is usually sparse. With the relaxed sparse constraint, the coefficient matrix C can be computed by solving an optimization problem as follows:

(1) $$\min\frac{1}{2\lambda }{\left\|X-XC\right\|}_{F}^{2}+{\left\|C\right\|}_{1}\;s.t.,diag\left(C\right)=0$$

where ${\left||\cdot |\right|}_{F}$ denotes the Frobenius norm which calculates the square root of sum of all squared elements, and constraint diag(C)=0 prevents the cells from being represented by themselves, while λ is a penalty factor. An efficient approach to solve Equation (1) is the alternating direction method of multipliers (ADMM) [23]. We rewrite Equation (1) as follows:

(2) $$\min\frac{1}{2\lambda }{\left\|X-XZ\right\|}_{F}^{2}+{\left\|C\right\|}_{1}\;s.t.,Z-C=0,diag\left(C\right)=0$$

where Z is an auxiliary matrix. According to the model of ADMM, the augmented Lagrangian with auxiliary matrix Z and penalty parameter (γ) > 0 for the optimization Equation (2) is

(3) $$L_{\frac{1}{\gamma }}\left(Z,C,Y\right)=\frac{1}{2\lambda }{\left\|X-XZ\right\|}_{F}^{2}+{\left\|C\right\|}_{1}+{\mathrm{tr}(Y}^{T}\left(Z-C\right))+\frac{1}{2\gamma }{\left\|C-Z\right\|}^{2}$$

where Y is the dual variable. The derivation of its update can be found in section 1 of File S1. Matrix C is the target sparse representation matrix. To keep the symmetry and nonnegative nature of similarity matrix, the element of SSR is calculated as ${\mathrm{sim}}_{\mathrm{sparse}}\left(i,j\right)=\left|c_{\mathrm{ij}}\right|+\left|c_{\mathrm{ji}}\right|$.

### Data preprocessing and gene selection

Before used to calculate SSR, the original data needs to be preprocessed. Various data preprocessing methods have been used in the previous studies, such as gene filtering [12], [16], feature selection [24], [25], and imputation [26], [27]. In this study, we first removed genes with zero expression in all of cells and applied $L_{2}$-norm to each cell to eliminate the expression scale difference between different cells. Then, we computed the preliminary SSR with the normalized gene expression matrix, and adopted the Laplacian score [28] on SSR to assess the contribution that genes make to cell-to-cell similarity learning. According to the Laplacian scores, we selected significant genes for the following study. Genes with higher Laplacian scores are considered as more informative in distinguishing cell types [8]. Besides the SSR, we also considered three additional pairwise similarities, *i.e.*, Pearson correlation, Spearman correlation, and cosine similarity, to evaluate the importance of genes (denoted as ${\mathrm{sim}}_{\mathrm{pearson}}$, ${\mathrm{sim}}_{\mathrm{spearman}}$, and ${\mathrm{sim}}_{\mathrm{cosine}}$, respectively). For each similarity, we ranked genes in descending order by the Laplacian score and selected the top t genes as an important gene set that is denoted by $G_{1}$. The determination of the threshold t can be formulated as

(4) $$\min\;var\;\left({\mathrm{LS}}_{G1}\right)+var\;\left({\mathrm{LS}}_{G2}\right)\;s.t.\;0.1\times p<\left|G_{1}\right|<0.5\times p$$

where $G_{1}$ = [$g_{1},g_{2},\cdots ,g_{t-1}$] and $G_{2}=\left[g_{t},g_{t+1},\cdots ,g_{p}\right]$ denote two gene sets divided by t. The ${\mathrm{LS}}_{G1}$ and ${\mathrm{LS}}_{G2}$ are the Laplacian scores of genes in sets $G_{1}$ and $G_{2}$, respectively, and $\left|*\right|$ is the cardinality of a set. The var(∗) indicates variance of a set while p is the number of genes. Finally, we recomputed ${\mathrm{sim}}_{\mathrm{sparse}}$, ${\mathrm{sim}}_{\mathrm{pearson}}$, ${\mathrm{sim}}_{\mathrm{spearman}}$, and ${\mathrm{sim}}_{\mathrm{cosine}}$ based on the intersection of four selected important gene sets. In the next section, we introduce an enhancement strategy to further improve the learned SSR ${\mathrm{sim}}_{\mathrm{sparse}}$.

### Similarity enhancement

The SSR ${\mathrm{sim}}_{\mathrm{sparse}}$ may suffer from the high-level technical noise in the data resulting in underestimation. Inspired by the consensus clustering and resource allocation, we further enhanced ${\mathrm{sim}}_{\mathrm{sparse}}$ by integrating multiple pairwise similarities including ${\mathrm{sim}}_{\mathrm{pearson}}$, ${\mathrm{sim}}_{\mathrm{spearman}}$, and ${\mathrm{sim}}_{\mathrm{cosine}}$. These pairwise similarities partially reveal the local information between cells.

We imputed the missing values in ${\mathrm{sim}}_{\mathrm{sparse}}$ according to their nearest neighbors’ information. We firstly defined a target similarity matrix P as follows:

(5) $$P\left(x_{i},x_{j}\right)=\left\{\begin{array}{l} 1,x_{j}\in KNN\left(x_{i}\right)\\ 0,else \end{array}\right)$$

where $KNN\left(x_{i}\right)$ indicates the KNN of cell $x_{i}$. Then we marked the similarity ${\mathrm{sim}}_{\mathrm{sparse}}\left(x_{i},x_{j}\right)$ between cells $x_{i}$ and $x_{j}$ as a missing value when it is zero in the ${\mathrm{sim}}_{\mathrm{sparse}}$ but $P\left(x_{i},x_{j}\right)=1$ in at least one pairwise similarity matrix. Let ${\mathrm{Isim}}_{\mathrm{sparse}}=O^{n\times n}$ denotes the initial matrix to be imputed where *n* indicates the number of cells. For a marked missing value, the similarity ${\mathrm{Isim}}_{\mathrm{sparse}}\left(x_{i},x_{j}\right)$ was computed by the modified Weighted Adamic/Adar [29], [30]. It was formulated as follows:

(6) $${\mathrm{Isim}}_{\mathrm{sparse}}\left(x_{i},x_{j}\right)=\sum_{x_{z}\in CN\left(x_{i},x_{j}\right)}\frac{{\mathrm{sim}}_{\mathrm{sparse}}\left(x_{i},x_{z}\right)+{\mathrm{sim}}_{\mathrm{sparse}}\left(x_{j},x_{z}\right)}{\left|\Gamma \left(x_{z}\right)\right|}$$

where $\left|\Gamma (x_{z})\right|$ indicates the number of neighbors of cell $x_{z}$, and $\mathrm{CN}(x_{i},x_{j})$ denotes the set of common neighbors of cell $x_{i}$ and $x_{j}$. Note that the imputed similarity ${\mathrm{Isim}}_{\mathrm{sparse}}\left(x_{i},x_{j}\right)$ is zero when $\mathrm{CN}(x_{i},x_{j})=\emptyset$. At the end, an enhanced and more comprehensive SSR matrix ${\mathrm{Esim}}_{\mathrm{sparse}}$ was computed as ${\mathrm{Esim}}_{\mathrm{sparse}}={\mathrm{Isim}}_{\mathrm{sparse}}+{{\mathrm{Isim}}_{\mathrm{sparse}}}^{T}$ + ${\mathrm{sim}}_{\mathrm{sparse}}$.

### Spectral clustering

Spectral clustering is a typical clustering technique that divides multiple objects into disjoint clusters depending on the spectrum of the similarity matrix [31]. Compared with the traditional clustering algorithms, spectral clustering is advantageous in model simplicity and robustness. In this study, we performed spectral clustering on the final enhanced SSR ${\mathrm{Esim}}_{\mathrm{sparse}}$. The inputs of spectral clustering are the cell-to-cell similarity matrix and the cluster number. The detailed introduction and analysis of spectral clustering could be found in previous studies [31], [32].

### Datasets

Datasets used in this study consist of two parts, real scRNA-seq datasets and simulated scRNA-seq datasets. We collected ten real scRNA-seq datasets that vary in terms of species, tissues, and biological processes, from public databases or published studies. The scale of these ten datasets varies from dozens to thousands, and the gene expression levels of them were computed by different units. The details of these real datasets are described in Table 1. Four datasets (*i.e.*, Treutlein [33], Deng [34], Ting [35], and Macosko [36] datasets) of these ten datasets were downloaded from the data subdirectory of MPSSC tool (https://github.com/ishspsy/project/tree/master/MPSSC). The Yan [37] and Goolam [38] datasets were collected from the popular single-cell consensus clustering (SC3) software package (https://github.com/hemberg-lab/SC3). The Song [39], Engel [40], and Haber [41] datasets were obtained via Gene Expression Omnibus [42] database (GEO: GSE85908, GSE74597, and GSE92332, respectively; https://www.ncbi.nlm.nih.gov/geo/), and the Vento [43] dataset was downloaded from ArrayExpress [44] (ArrayExpress: E-MTAB-6678; https://www.ebi.ac.uk/arrayexpress/). In addition, we used Splatter [45] to simulate five scRNA-seq datasets for more comprehensive analysis. They either have different size or different sparsity. We set *group.prob* to (0.65, 0.25, 0.1) for all simulated datasets, and changed the scale and sparsity by adjusting *nCells* and *dropout.mid*, respectively. The other parameters were set to default. The sample sizes of the five simulated datasets are 1000, 1000, 1000, 500, and 1500, and the corresponding sparsity is 0.61, 0.8, 0.94, 0.94, and 0.94, respectively.

**Table 1:** **The details of real scRNA-seq datasets used in this study**




*Note*: FPKM, fragments per kilobase of exon model per million mapped fragments; RPKM, reads per kilobase of exon model per million mapped reads; CPM, counts of exon model per million mapped reads; RPM, reads of exon model per million mapped reads; TPM, transcripts per kilobase of exon model per million mapped reads; UMI, unique molecular identifier.

### scRNA-seq clustering methods

For performance comparison, we took the original SSR, native spectral clustering (SC), and eight state-of-the-art clustering methods (*i.e.*, SIMLR [8], MPSSC [20], Corr [11], SNN-Cliq [10], NMF [13], SC3 [16], dropClust [46], and Seurat [47]) as comparison. Among these methods, SIMLR, MPSSC, Corr, and SNN-Clip focus on similarity learning. Both SIMLR and MPSSC learn a representative similarity matrix from multi-Gaussian-kernels with different resolutions. Corr introduces a cell-pair differentiability correlation to relieve the effect of dropouts. SNN-Cliq applies the SNN to redefine the pairwise similarity. NMF detects the type of cells by projecting the high dimensional data into a latent space, in which each dimension of the latent space denotes a specific type. SC3 is a typical and powerful consensus clustering method. It obtains clusters by applying different upstream processes, and desires the final clusters to fit better. DropClust is a clustering algorithm designed for large-scale single-cell data, and it exploits an approximate nearest neighbor search technique to reduce the time complexity of analyzing large-scale data. Seurat, a popular R package for single-cell data analysis, obtains cell groups based on KNN-graph and Louvain clustering. Moreover, SC [32] with the Pearson correlation is considered as a baseline.

### Metric of performance evaluation

We evaluated the proposed approach using two common metrics, *i.e.*, normalized mutual information (NMI) [48] and adjusted rand index (ARI) [49]. They have been widely used to assess clustering performance. Both NMI and ARI evaluate the consistency between the obtained clustering and pre-annotated labels, and have slightly different emphasis [50]. Given the real labels L1 and the clustering labels L2, NMI is calculate as

(7) $$NMI\left(L1,L2\right)=\frac{I(L1,L2)}{[H\left(L1\right)+H\left(L2\right)]/2}$$

I(L1,L2) is the mutual information between L1 and L2, and *H* denotes entropy. For ARI, given L1 and L2, it is computed as

(8) $$ARI\left(L1,L2\right)=\frac{\sum_{\mathrm{ij}}\left(\frac{n_{\mathrm{ij}}}{2}\right)-[\sum_{\mathrm{ij}}\left(\frac{n_{\mathrm{ij}}}{2}\right)\sum_{\mathrm{ij}}\left(\frac{n_{\mathrm{ij}}}{2}\right)]/\left(\frac{n}{2}\right)}{\frac{1}{2}\left[\sum_{i}\left(\frac{a_{i}}{2}\right)+\sum_{j}\left(\frac{b_{j}}{2}\right)\right]-\left[\sum_{i}\left(\frac{a_{i}}{2}\right)\sum_{j}\left(\frac{b_{j}}{2}\right)\right]/\left(\frac{n}{2}\right)}$$

where $n_{\mathrm{ij}}$ is the number of cells in both group ${L1}_{i}$ and group ${L2}_{j}$. The $a_{i}$ and $b_{j}$ denote the number of cells in group ${L1}_{i}$ and group ${L2}_{j}$, respectively.

## Results and discussion

### SSRE can greatly improve the clustering accuracy

In order to evaluate the performance of SSRE comprehensively, we first applied it on ten pre-annotated real scRNA-seq datasets and compared its performance with the original SSR, SC, and eight state-of-the-art clustering methods. See details in the Method section. Then, we tested all these methods on five simulated datasets for further comparison. In our experiments, for a fair comparison, we set the number of clusters to the number of pre-annotated types for all methods except SNN-Cliq and Seurat because SNN-Cliq and Seurat do not need the number of clusters as input. The other parameters in all the methods were set to the default as described in the original papers. Table 2 and Table 3 summarize the NMI and ARI values of all methods on ten real scRNA-seq datasets, respectively. The results of Corr in large datasets are unreachable because of the high computational complexity. As shown in Table 2 and Table 3, the proposed method SSRE outperformed all other methods in most cases. SSRE achieved the best or tied first on seven datasets upon NMI and ARI. Meanwhile, SSRE ranked the second on three datasets based on NMI and two datasets based on ARI. It demonstrates that SSRE obtains more reliable results independent to the scale and the biological conditions of scRNA-seq data. Moreover, SSRE performed better than SSR on nine of the ten datasets in terms of NMI and ARI, which illustrates the effectiveness of the enhancement strategy in SSRE. Results of simulation experiment are shown in Tables S1 and S2. SSRE achieved the better performance overall, which shows the good stability of SSRE. SSRE is slightly time-consuming compared with some methods such as SC and dropClust, but its running time is still in a reasonable range. More detailed descriptions can be found in section 2 of File S1.

**Table 2:** **NMI values of all analyzed methods across ten real datasets**




*Note*: SC, native spectral clustering; SNN, shared nearest neighbor; SIMLR, single-cell interpretation via multikernel learning; SC3, single-cell consensus clustering; NMF, nonnegative matrix factorization; SSR, sparse subspace representation; SSRE, single-cell clustering framework based on similarity learning. “–” indicates unreachable. The bold value is the highest value in each column.

**Table 3:** **ARI values of all analyzed methods across ten real datasets**




*Note*: The bold value is the highest value in each column.

Estimating number of clusters is another key step in most clustering methods, which affects the accuracy of clustering method. SSRE performed eigengap [32] on the learned similarity matrix to estimate the number of clusters. Eigengap is a typical cluster number estimation method. It determines the number of clusters by calculating max gap between eigenvalues of a Laplacian matrix. To assess reliability of the estimation in different methods, we compared the estimated numbers with pre-annotated numbers. The results are summarized in Table S3. Besides SSRE and SSR, another four methods which also focus on similarity learning were selected for comparison. More experimental details can be seen in section 3 of File S1.

### Analysis of parameter setting

**Figure 2:**
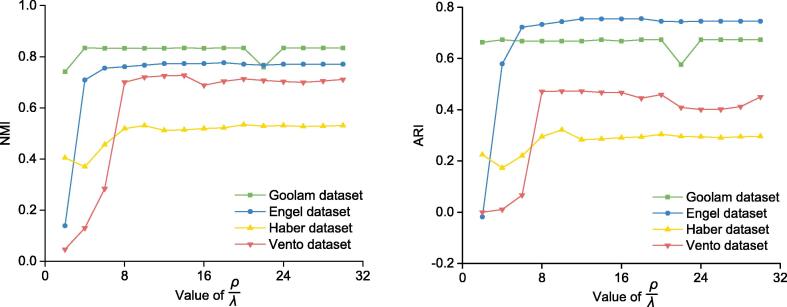
**Clustering performance of SSRE with different parameter settings** The change of clustering performance of SSRE *versus* the value of parameter ρ/λ on four datasets (*i.e.*, Goolam dataset [38], Engel dataset [40], Haber dataset [41], and Vento dataset [43]) is shown here. The change of NMI values (**A**) and ARI values (**B**). NMI, normalized mutual information; ARI, adjusted rand index.

In SSRE, four parameters are required to be set by users, *i.e.*, penalty coefficients λ and γ in solving SSR ${\mathrm{sim}}_{\mathrm{sparse}}$, gene selection threshold t, and the number of nearest neighbors k in similarity enhancement procedure. In this study, the selection of the threshold t was determined adaptively by solving Equation (4). The number of nearest neighbors k was set to 0.1×n (n is the number of cells) for small datasets with less than 5000 cells and set to 100 for other larger datasets. The other two parameters λ and γ in augmented Lagrangian (we used 1/λ and 1/γ in the coding implementation) were proportionally set as:

(9) $$1/\gamma =\rho /\lambda ,\rho =\min_{j}\left\{\max_{i}\left\{m_{\mathrm{ij}}\right\}\right\}$$

where $m_{\mathrm{ij}}$ is the element of matrix $M=X^{T}X$. The $m_{\mathrm{ij}}$ is equivalent to the cosine similarity between cells $x_{i}$ and $x_{j}$. This is same as previous work [19]. In our experiments, ρ/λ was set to a constant. For a given dataset, the larger value of ρ leads to the larger value of λ, which will result in the sparser matrix *C*. It means that the value of ρ can control the sparsity of matrix *C* adaptively in different datasets. Moreover, to validate the effect of penalty coefficient λ in clustering results, we tested SSRE with ρ/λ from 2 to 30 with the increment of 2 on all real datasets. We found that SSRE’s performance was basically stable when ρ/λ is in the interval of 6 and 20. The results are shown in Figure 2 and Figure S1. In our study, we set ρ/λ to 10 and 1/λ=ρ/λ as default for all datasets.

### Application of SSRE in visualization

**Figure 3:**
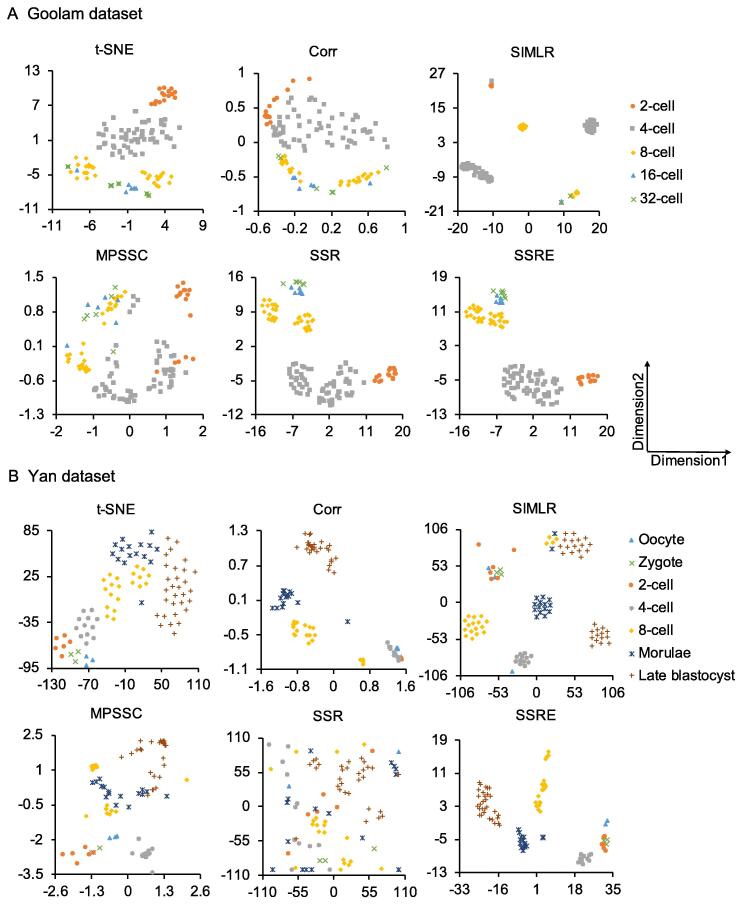
**Visualization of Goolam and Yan datasets using different methods** Two datasets are visualized by t-SNE, Corr, SIMLR, MPSSC, SSR, and SSRE, respectively. **A.** The clustering results from Goolam dataset [38]. **B.** The clustering results from Yan dataset [37]. Each point in the figure represents a cell. Different colors and shapes indicate different cell types. t-SNE, t-distributed stochastic neighbor embedding; SIMLR, single-cell interpretation via multikernel learning.

One of the most valuable aims in single-cell analysis is to identify new cell types or subtypes [6]. Visualization is an effective tool to intuitively display subgroups of all cells. The t-distributed stochastic neighbor embedding (t-SNE) [51] is one of the most popular visualization methods, and it has been proved to be powerful in scRNA-seq data. In our study, we performed a modified t-SNE on the similarities learned by different methods for visualization. We focused on two datasets, Goolam and Yan, and selected the native t-SNE, Corr, SIMLR, MPSSC, SSR, and SSRE for comparison. In Goolam dataset [38], cells were derived from mouse embryos in five differentiation stages: 2-cell, 4-cell, 8-cell, 16-cell, and 32-cell. The visualization results of Goolam dataset are shown in Figure 3A. As shown in Figure 3A, SSRE placed cells with the same type together and distinguished cells with different types clearly. And, although SIMLR can clearly distinguish groups from each other, some cells with the same type were separated. The second dataset Yan [37] was obtained from human pre-implantation embryos. It involves seven primary stages of preimplantation development: metaphase II oocyte, zygote, 2-cell, 4-cell, 8-cell, morula, and late blastocyst. As shown in Figure 3B, Corr, SIMLR, and SSRE had a better overall performance than other methods. However, the four cell types, *i.e.*, oocyte, zygote, 2-cell, and 4-cell, were mixed totally in Corr, and mixed partially in SIMLR. Moreover, SIMLR also divided the cells with same type into different groups that were generally far away from each other. SSRE clusters cells more accurately, according to oocyte, 2-cell, and other cell types, than the competing methods.

### Application of SSRE in identifying differentially expressed genes

The predicted clusters may potentially enable enhanced downstream scRNA-seq data analysis in biological sights. As a demonstration, we aimed to detect significantly differentially expressed genes (DEGs) based on the clustering results. Specifically, we applied the Kruskal-Wallis test [52] to the gene expression profiles with the inferred labels. The Kruskal-Wallis test, a non-parametric method, is often used for testing that if two or more groups are from the same distribution. We used the R function kruskal.test to perform the Kruskal-Wallis test. Then we detected DEGs according to the *P* value. The significant *P* value (*P* < 0.01) of a gene indicates that the gene’s expression in at least one group stochastically dominates one other group. We took the Yan [37] dataset as an example to analyze the DEGs. The details of Yan have been introduced above. Figure S2 shows the heat map of gene expression of the top 50 most significantly DEGs identified. Notice that genes *NLRP11*, *NLRP4*, *CLEC10A*, *H1FOO*, *GDF9*, *OTX2*, *ACCSL*, *TUBB8*, and *TUBB4Q* have been reported in previous studies [37], [53], which were also identified by SSRE. Genes *CLEC10A*, *H1FOO*, and *ACCSL* were reported as the markers of 1-cell stage cells (zygote) of human early embryos, while *NLRP11* and *TUBB4Q* are the markers of 4-cell stage cells [54]. Genes *GDF9* and *OTX2* are the markers of germ cell and primitive endoderm cell, respectively [55], [56]. Genes *H1FOO* and *GDF9* were marked as the potential stage-specific genes in the oocyte and the blastomere of 4-cell stage embryos [57]. Certain *PRAMEF* family genes were reported as ones with transiently enhanced transcription activity in 8-cell stage. *MBD3L* family genes were identified as 8-cell stage-specific genes during the human embryo development in the previous studies [58], [59]. All these are part of the top 50 significantly DEGs detected by SSRE.

## Conclusion

Identifying cell types from single-cell transcriptome data is a meaningful but challengeable task because of the high-level noise and high dimension. The ideal identification of cell types enables more reliable characterizations of a biological process or phenomenon. Otherwise, it will introduce additional biases. Many approaches from different perspectives have been proposed recently, but the accuracy of cell type identification is still far from expectation. In this study, we presented SSRE, a similarity learning-based computational framework for cell type identification. Besides three classical pairwise similarities, SSRE computed the SSR of cells based on the subspace theory. Moreover, a gene selection process and an enhancement strategy were designed based on the characteristics of different similarities to learn more reliable similarities. SSRE greatly improved the clustering performance by appropriately combining multiple similarity measurements and adopting the embedding of sparse structure. The systematic performance evaluations on multiple scRNA-seq datasets showed that SSRE achieves superior performance among all competing methods. Furthermore, with the further downstream analyses, it is demonstrated that the learned similarity and inferred clusters can potentially be applied to more exploratory analyses, such as identifying gene markers and detecting new cell subtypes. In addition, for more flexible use, users can choose one or two of the three pairwise similarities mentioned in this study to perform gene selection and similarity enhancement procedures, and all three are used by default. Nonetheless, the proposed computational framework still can be improved in future study. One limitation of SSRE is relatively time-consuming in large-scale datasets; therefore, parallel computing is a possible strategy to accelerate the framework [60]. And more informative genes can be extracted or other biological information, such as gene functions [61] and gene regulatory relationships [62], [63], can be incorporated to distinguish cell types. In addition, with the emergence of single-cell multi-omics data, it will be a possible trend to design corresponding multi-view clustering models to integrate the multi-omics data for cell type identification [64], [65].

## Code availability

The matlab and python implementations of SSRE are available at https://github.com/CSUBioGroup/SSRE.

## CRediT author statement

**Zhenlan Liang:** Conceptualization, Methodology, Validation, Writing - original draft. **Min Li:** Supervision, Methodology, Writing - review & editing. **Ruiqing Zheng:** Methodology, Writing - original draft. **Yu Tian:** Data curation, Validation. **Xuhua Yan:** Software. **Jin Chen:** Writing - review & editing. **Fang-Xiang Wu:** Writing - review & editing. **Jianxin Wang:** Writing - review & editing. All authors read and approved the final manuscript.

## Competing interests

The authors have declared that they have no competing interests.

## Supplementary materials

Supplementary File S1 **The ADMM used in SSRE and performance assessment of SSRE**

Supplementary Figure S1 **Clustering performance of SSRE with different parameter settings** The change of clustering performance of SSRE versus the value of parameter ρ/λ on six datasets (i.e., Treutlein dataset [33], Yan dataset [37], Deng dataset [34], Ting dataset [35], Song dataset [39], Macosko dataset [36]) is shown here. The change of NMI values **(A)** and ARI values **(B)**.

Supplementary Figure S2 **Top 50 DEGs of Yan dataset identified by SSRE** The heat map of top 50 most significant DEGs identified by SSRE on Yan dataset [37]. These genes are ranked according to the P values. The P values of all these 50 genes are small (Kruskal-Wallis test P < 2.5E-11). Each row indicates a gene and each column indicates a cell. Colors on the top annotation and cell type legend correspond to seven pre-annotated cell types. The pre-annotated cell types are provided by the original paper of Yan dataset. Colors on the gene expression legend correspond to different gene expression levels. RPKM, reads per kilobase of exon model per million mapped reads; DEGs, differentially expressed genes.
