# Supplementary material for: SSRE: Cell Type Detection Based on Sparse Subspace Representation and Similarity Enhancement
Source: Genomics Proteomics Bioinformatics. 2021 Feb 27;19(2):282–91. doi: 10.1016/j.gpb.2020.09.004 (PMC8602764; doi:10.1016/j.gpb.2020.09.004)
Supplement: Supplementary File S1 — The ADMM used in SSRE and performance assessment of SSRE [file mmc1.docx]

File S1 The ADMM used in SSRE and performance assessment of SSRE

Section 1: The ADMM used in SSRE

According to the optimization program (2) in the main context, the augmented Lagrangian formulation is written as follows:

$$\mathcal{L}_{\frac{1}{\gamma}}\left( Z,C,Y \right)=\frac{1}{2\lambda} \left\| X-XZ \right\|_{F}^{2}+\left\| \left. C \right\| \right._{1}+tr(Y^{T}\left( Z-C \right))+\frac{1}{2\gamma}\left\| \left. C-Z \right\| \right.^{2} (S1)$$

where *X* is the gene expression matrix, *C* is the target sparse representation matrix to be solved, *J* is an auxiliary matrix, *Y* is the dual variable or Lagrange multiplier and $\gamma$ is a user-defined parameter. The ADMM updates the one of matrix *C*, *Z*, *Y* by fixing others each time with the following formulas

$Z^{k+1}=\mathrm{argmin}_{Z} \mathcal{L}_{\frac{1}{\gamma}}\left( Z,C^{k},Y^{k} \right) (S2)$

$=\mathrm{argmin}_{Z}\left\{ \frac{1}{2\lambda} \left\| X-XZ \right\|_{F}^{2}+\left\| C^{k} \right\|_{1}+tr\left( {Y^{k}}^{T}\left( Z-C^{k} \right) \right)+\frac{1}{2\gamma}\left\| \left. C^{k}-Z \right\| \right.^{2} \right\}$

$=\left( \frac{X^{T}X}{\lambda}+\frac{1}{\gamma} \right)^{-1}(\frac{X^{T}X}{\lambda}-{Y^{k}}^{T}+\frac{1}{\gamma}C^{k}$)

$$C^{k+1}=\mathrm{argmin}_{C} \mathcal{L}_{\frac{1}{\gamma}}\left( Z^{k+1},C,Y^{k} \right) \left( S3 \right)$$

$$=\mathrm{argmin}_{C}\left\{ \frac{1}{2\lambda} \left\| X-XZ^{k+1} \right\|_{F}^{2}+\left\| \left. C \right\| \right._{1}+tr\left( {Y^{k}}^{T}\left( Z^{k+1}-C \right) \right) +\frac{1}{2\gamma}\left\| \left. C-Z^{k+1} \right\| \right.^{2} \right\}$$

$={Soft}_{\lambda,\gamma}\left( Z^{k+1}+\gamma Y^{k} \right)$

$$Y^{k+1}= Y^{k}+\frac{1}{\gamma}(Z^{k+1}-C^{k+1}) \left( S4 \right)$$

where ${Soft}_{\lambda,\gamma}\left( * \right)$ is a soft-thresholding operator [[1](#_ENREF_1)].

Section 2: Results on simulated datasets

Besides real datasets, we also apply SSRE on five simulated datasets with different size and sparsity. The size and sparsity of five simulated datasets are as: Sim_data_1 (size: 1000cells, sparsity:0.61), Sim_data_2 (size: 1000cells, sparsity:0.8), Sim_data_3 (size: 1000cells, sparsity:0.94), Sim_data_4 (size: 500cells, sparsity: 0.94), Sim_data_5 (size: 1500cells, sparsity:0.94). Table S1 and Table S2 summarize the results on datasets with different sparsity and different size respectively, where the results of Corr in Sim_data_5 is unreachable because of high computational complexity. We can see that SSRE has the best performance overall in two simulation experiments in terms of NMI and ARI. DropClust gets the smallest running time on almost all datasets, but the running time needed by SSRE is also acceptable

Section 3: The estimation of number of clusters

With the learned similarity of SSR and SSRE, we apply the eigengap [[2](#_ENREF_2)] to estimate the number of clusters. Moreover, we choose SIMLR [[3](#_ENREF_3)], MPSSC [[4](#_ENREF_4)], Corr [[5](#_ENREF_5)], and SNN-Cliq [[6](#_ENREF_6)] as the competing methods which also focus on similarity learning. For these compared methods, we perform the corresponding estimation algorithm provided by themselves. Table S3 summarizes the results on ten real datasets. It shows that the proposed method SSRE gets the same number as pre-annotated number in two datasets (Ting [[7](#_ENREF_7)] and Vento [[8](#_ENREF_8)]) and closest with pre-annotated number in most datasets (Treutlein [[9](#_ENREF_9)], Goolam [[10](#_ENREF_10)], Song [[11](#_ENREF_11)], Haber [[12](#_ENREF_12)] and Macosko [[13](#_ENREF_13)]). We can see that there is no method can estimate the number of clusters as same as pre-annotated numbers in all datasets, and SSRE has a better performance overall.

References

[1] Cai J-F, Candès EJ, Shen Z. A singular value thresholding algorithm for matrix completion. SIAM J Optim 2010;20:1956–82.

[2] Von Luxburg U. A tutorial on spectral clustering. Stat Comput 2007;17:395–416.

[3] Wang B, Zhu J, Pierson E, Ramazzotti D, Batzoglou S. Visualization and analysis of single-cell RNA-seq data by kernel-based similarity learning. Nat Methods 2017;14:414–6.

[4] Park S, Zhao H. Spectral clustering based on learning similarity matrix. Bioinformatics 2018;34:2069–76.

[5] Jiang H, Sohn L, Huang H, Chen L. Single Cell Clustering Based on Cell-Pair Differentiability Correlation and Variance Analysis. Bioinformatics 2018; 34:3684–94.

[6] Xu C, Su Z. Identification of cell types from single-cell transcriptomes using a novel clustering method. Bioinformatics 2015;31:1974–80.

[7] Ting DT, Wittner BS, Ligorio M, Jordan NV, Shah AM, Miyamoto DT, et al. Single-cell RNA sequencing identifies extracellular matrix gene expression by pancreatic circulating tumor cells. Cell reports 2014;8:1905–18.

[8] Vento-Tormo R, Efremova M, Botting RA, Turco MY, Vento-Tormo M, Meyer KB, et al. Single-cell reconstruction of the early maternal–fetal interface in humans. Nature 2018;563: 347–53.

[9] Treutlein B, Brownfield DG, Wu AR, Neff NF, Mantalas GL, Espinoza FH, et al. Reconstructing lineage hierarchies of the distal lung epithelium using single-cell RNA-seq. Nature 2014;509:371–5.

[10] Goolam M, Scialdone A, Graham SJ, Macaulay IC, Jedrusik A, Hupalowska A, et al. Heterogeneity in Oct4 and Sox2 targets biases cell fate in 4-cell mouse embryos. Cell 2016;165:61–74.

[11] Song Y, Botvinnik OB, Lovci MT, Kakaradov B, Liu P, Xu JL, et al. Single-cell alternative splicing analysis with expedition reveals splicing dynamics during neuron differentiation. Mol Cell 2017;67:148–61.

[12] Haber AL, Biton M, Rogel N, Herbst RH, Shekhar K, Smillie C, et al. A single-cell survey of the small intestinal epithelium. Nature 2017;551:333–9.

[13] Macosko EZ, Basu A, Satija R, Nemesh J, Shekhar K, Goldman M, et al. Highly parallel genome-wide expression profiling of individual cells using nanoliter droplets. Cell 2015;161:1202–14.
