# Supplementary figures and images for: SSRE: Cell Type Detection Based on Sparse Subspace Representation and Similarity Enhancement
Source: Genomics Proteomics Bioinformatics. 2021 Feb 27;19(2):282–91. doi: 10.1016/j.gpb.2020.09.004 (PMC8602764; doi:10.1016/j.gpb.2020.09.004)

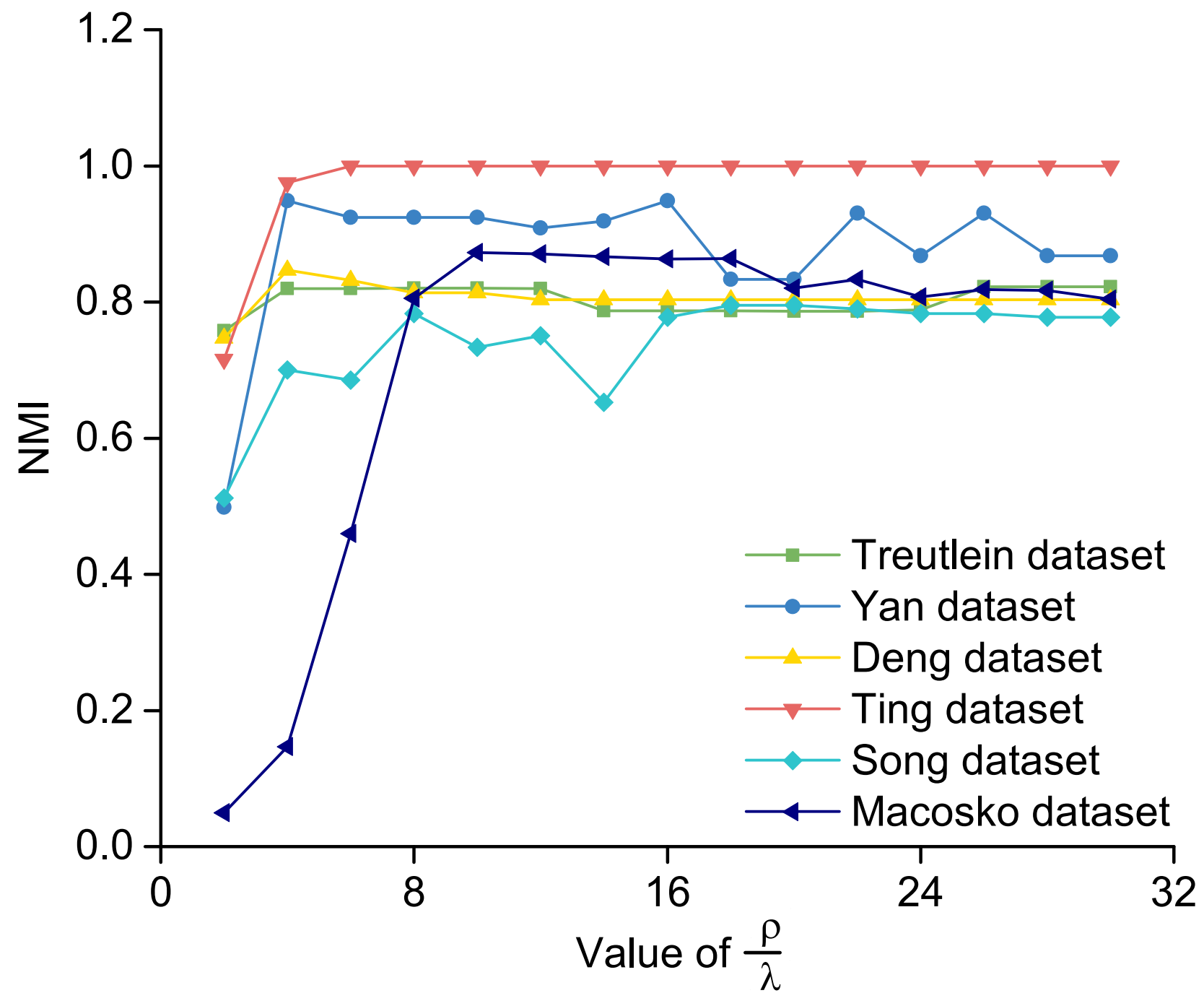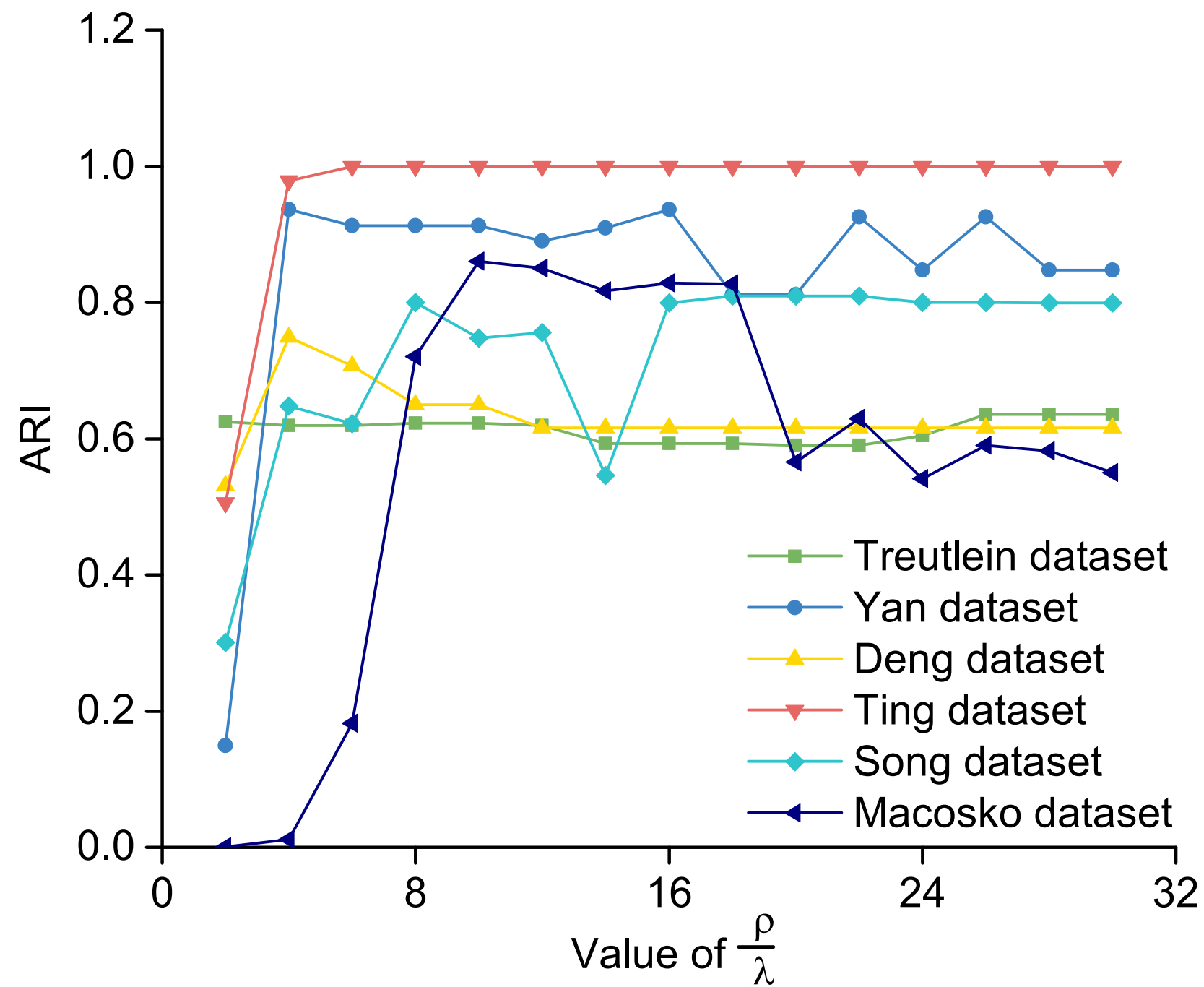

Supplement: Supplementary Figure S1 — Clustering performance of SSRE with different parameter settings The change of clustering performance of SSRE versus the value of parameter ρ/λ on six datasets (i.e., Treutlein dataset [33], Yan dataset [37], Deng dataset [34], Ting dataset [35], Song dataset [39], Macosko dataset [36]) is shown here. The change of NMI values (A) and ARI values (B). [file mmc2.pdf]

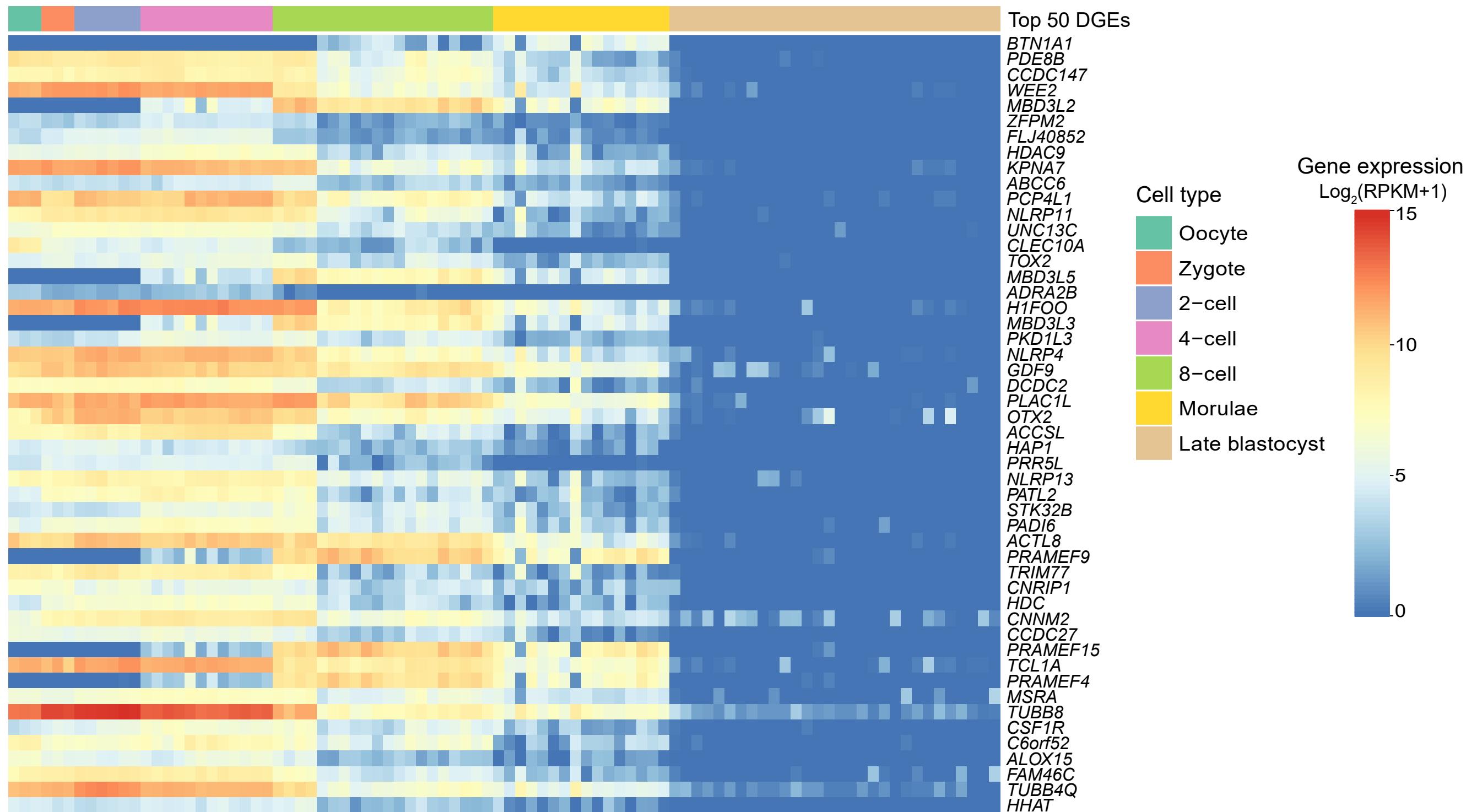

Supplement: Supplementary Figure S2 — Top 50 DEGs of Yan dataset identified by SSRE The heat map of top 50 most significant DEGs identified by SSRE on Yan dataset [37]. These genes are ranked according to the P values. The P values of all these 50 genes are small (Kruskal-Wallis test P < 2.5E-11). Each row indicates a gene and each column indicates a cell. Colors on the top annotation and cell type legend correspond to seven pre-annotated cell types. The pre-annotated cell types are provided by the original paper of Yan dataset. Colors on the gene expression legend correspond to different gene expression levels. RPKM, reads per kilobase of exon model per million mapped reads; DEGs, differentially expressed genes. [file mmc3.pdf]
