# Supplementary material for: SSRE: Cell Type Detection Based on Sparse Subspace Representation and Similarity Enhancement
Source: Genomics Proteomics Bioinformatics. 2021 Feb 27;19(2):282–91. doi: 10.1016/j.gpb.2020.09.004 (PMC8602764; doi:10.1016/j.gpb.2020.09.004)
Supplement: Supplementary Table S1 [file mmc4.docx]

**Table S1** **Results of all analyzed methods on simulated datasets with different sparsity**

| **Method** | **Sim_data_1** | | | **Sim_data_2** | | | **Sim data 3** | | |
| --- | --- | --- | --- | --- | --- | --- | --- | --- | --- |
|  | **NMI** | **ARI** | **Time (s)** | **NMI** | **ARI** | **Time (s)** | **NMI** | **ARI** | **Time (s)** |
| SC | 0.98 | 0.99 | 8.79 | 0.95 | 0.97 | 7.68 | 0.61 | 0.63 | 7.34 |
| SNN-Cliq | 0.43 | 0.03 | 24.21 | 0.42 | 0.03 | 25.58 | 0.28 | 0.01 | 25.54 |
| SIMLR | 0.98 | 0.99 | 49.76 | 0.97 | 0.99 | 48.70 | 0.42 | 0.35 | 32.55 |
| SC3 | **1.00** | **1.00** | 1404.4 | **0.99** | **1.00** | 1323.1 | 0.67 | 0.69 | 1393.0 |
| NMF | 0.96 | 0.98 | 403.28 | 0.93 | 0.96 | 183.27 | 0.62 | 0.67 | 55.03 |
| MPSSC | 0.48 | 0.41 | 29.63 | 0.53 | 0.43 | 84.31 | 0.39 | 0.36 | 26.87 |
| Corr | 0.64 | 0.75 | 37,144 | 0.59 | 0.70 | 35,300 | 0.02 | -0.01 | 34,302 |
| dropClust | 0.80 | 0.65 | **8.78** | 0.76 | 0.57 | **6.90** | 0.45 | 0.41 | **6.14** |
| Seurat | 0.73 | 0.52 | 18.03 | 0.81 | 0.61 | 16.95 | 0.66 | 0.55 | 15.58 |
| SSR | **1.00** | **1.00** | 35.56 | **0.99** | **1.00** | 43.92 | 0.65 | 0.72 | 46.26 |
| SSRE | **1.00** | **1.00** | 65.99 | **0.99** | 0.99 | 59.44 | **0.69** | **0.80** | 56.32 |

*Note*: Sim data 1 (size: 1000 cells, sparsity: 0.61), Sim data 2 (size: 1000 cells, sparsity: 0.8), Sim data 3 (size: 1000 cells, sparsity: 0.94).
