# Supplementary material for: SSRE: Cell Type Detection Based on Sparse Subspace Representation and Similarity Enhancement
Source: Genomics Proteomics Bioinformatics. 2021 Feb 27;19(2):282–91. doi: 10.1016/j.gpb.2020.09.004 (PMC8602764; doi:10.1016/j.gpb.2020.09.004)
Supplement: Supplementary Table S2 [file mmc5.docx]

**Table S2 Results of all analyzed methods on simulated datasets with different sizes**

| **Method** | **Sim_data_4** | | | **Sim_data_3** | | | **Sim data 5** | | |
| --- | --- | --- | --- | --- | --- | --- | --- | --- | --- |
|  | **NMI** | **ARI** | **Time (s)** | **NMI** | **ARI** | **Time (s)** | **NMI** | **ARI** | **Time (s)** |
| SC | 0.68 | 0.71 | **2.98** | 0.61 | 0.63 | 7.34 | 0.38 | 0.37 | 12.50 |
| SNN-Cliq | 0.35 | 0.02 | 7.00 | 0.28 | 0.01 | 25.54 | 0.22 | 0.01 | 61.63 |
| SIMLR | 0.46 | 0.38 | 12.66 | 0.42 | 0.35 | 32.55 | 0.31 | 0.30 | 78.93 |
| SC3 | **0.95** | **0.98** | 858.5 | 0.67 | 0.69 | 1393.0 | 0.42 | 0.54 | 3075.8 |
| NMF | 0.78 | 0.83 | 43.25 | 0.62 | 0.67 | 55.03 | 0.46 | 0.54 | 73.03 |
| MPSSC | 0.57 | 0.43 | 5.10 | 0.39 | 0.36 | 26.87 | 0.44 | 0.37 | 338.10 |
| Corr | 0.02 | 0.01 | 5123.10 | 0.02 | -0.01 | 34,302 | - | - | - |
| dropClust | 0.64 | 0.50 | 3.18 | 0.45 | 0.41 | **6.14** | 0.31 | 0.24 | **8.84** |
| Seurat | 0.68 | 0.57 | 14.83 | 0.66 | 0.55 | 15.58 | **0.76** | 0.63 | 18.64 |
| SSR | 0.92 | 0.96 | 11.60 | 0.65 | 0.72 | 46.26 | 0.57 | 0.65 | 79.78 |
| SSRE | **0.95** | **0.98** | 13.92 | **0.69** | **0.80** | 56.32 | 0.63 | **0.72** | 103.36 |

*Note*: Sim data 4 (size: 500 cells, sparsity: 0.94), Sim data 3 (size: 1000 cells, sparsity: 0.94, Sim data 5 (size: 1500 cells, sparsity: 0.94).
