# Supplementary material for: SSRE: Cell Type Detection Based on Sparse Subspace Representation and Similarity Enhancement
Source: Genomics Proteomics Bioinformatics. 2021 Feb 27;19(2):282–91. doi: 10.1016/j.gpb.2020.09.004 (PMC8602764; doi:10.1016/j.gpb.2020.09.004)
Supplement: Supplementary Table S3 [file mmc6.docx]

**Table S3 The number of clusters estimated by different methods**

| **Dataset** | **Pre-annotated number** | **SSRE** | **SSR** | **SNN-Cliq** | **Corr** | **SIMLR** | **MPSSC** |
| --- | --- | --- | --- | --- | --- | --- | --- |
| Treutlein | 5 | 4 | 4 | 14 | 3 | 10 | 15 |
| Yan | 7 | 14 | 16 | 18 | 5 | 12 | 16 |
| Deng | 7 | 5 | 5 | 8 | 2 | 9 | 17 |
| Goolam | 5 | 3 | 8 | 17 | 3 | 11 | 15 |
| Ting | 5 | 5 | 5 | 8 | 3 | 5 | 15 |
| Song | 4 | 3 | 4 | 30 | 2 | 3 | 14 |
| Engel | 4 | 1 | 1 | 13 | 2 | 3 | 2 |
| Haber | 9 | 8 | 15 | 301 | - | 10 | 15 |
| Vento | 38 | 38 | 40 | 178 | - | 11 | 1 |
| Macosko | 39 | 42 | 49 | 548 | - | 6 | 11 |
